# Identifying Correlations between Chromosomal Proximity of Genes and Distance of Their Products in Protein-Protein Interaction Networks of Yeast

**DOI:** 10.1371/journal.pone.0057707

**Published:** 2013-03-06

**Authors:** Daniele Santoni, Filippo Castiglione, Paola Paci

**Affiliations:** 1 Institute for System Analysis and Computer Science “Antonio Ruberti”, National Research Council of Italy, Rome, Italy; 2 Institute for Computing Applications “Mauro Picone”, National Research Council of Italy, Rome, Italy; Kyushu Institute of Technology, Japan

## Abstract

In this article we present evidence for a relationship between chromosome gene loci and the topological properties of the protein-protein interaction network corresponding to the set of genes under consideration. Specifically, for each chromosome of the *Saccharomyces cerevisiae* genome, the distribution of the intra-chromosome inter-gene distances was analyzed and a positive correlation with the distance among the corresponding proteins of the protein-protein interaction network was found. In order to study this relationship we used concepts based on non-parametric statistics and information theory.

We provide statistical evidence that if two genes are closely located, then it is likely that their protein products are closely located in the protein-protein interaction network, or in other words, that they are involved in the same biological process.

## Introduction

Coexpression of neighboring genes is commonly found in prokaryotes due to transcriptional operons. Although eukaryotes do not have such transcriptional machinery, it has been recently highlighted that also in those organisms, neighboring genes have a higher index of positive coexpression than those predicted by chance [1], [2]. This pattern was observed for all eukaryotic genomes studied so far. However it is still unclear whether this is due to selective influences or not [1]. Several studies focused on eukaryotic genomes, investigated the relationship between chromosomal proximity of genes and their level of coexpression. For example, Kruglyak and Tang [1], [2], through the analysis of gene expression data in *Saccharomyces cerevisiae* observed the existence of a multi-gene regulatory mechanism reminding, to some extent, transcriptional operons, providing a list of candidate gene pairs that are likely to be controlled with this mechanism. Moreover Lee and Sonnhammer [2] focused on genomic clustering of genes of a pathway and hypothesized the coregulation of sets of functionally cooperating genes. The correlation between gene proximity and function was already observed in multiple bacterial genomes [3].

Recent experiments on mRNA molecules indicate that chromatin modification might provide a rationale for the coexpression of neighbor genes [4]. Batada et al. [5] investigated the relationship in genomic domains between coexpression rate and nucleosome occupancy, showing that higher nucleosome occupancy implies higher gene coexpression. These results support the hypothesis that frequently fluctuating chromatin, corresponding to high nucleosome occupancy, favors coexpression. The authors then suggest that selection might act in increasing coexpression level and contribute to the conservation of gene pair [5].

Following this view it is evident that the expression “chromatin structure” involves a wide range of phenomena and levels of organization, from the atomic details to larger scales. Understanding the local organization of nucleosomes is critical for understanding how chromatin impacts gene regulation.

The synthesis of proteins within a cell is a complex multi-step process encompassing different activities starting with the transcription of a gene and terminating with the translation into a protein. In the transcription of genes, located on different DNA segments, the structure of the chromatin plays a pivotal role, allowing the polymerase to access the various nucleotide regions [6]. A large portion of the genome in eukaryotic organisms is organized in compact compartments according to the three dimensional structure of the chromosome [7].

The basic unit in the packaging process is the nucleosome, the DNA-protein complex that folds in higher order structures, compacting DNA more than 10 000 times in an arrangement called chromatin. Despite its extremely compact structure the DNA must be accessible to the protein machineries allowing biological processes such as replication, transcription, recombination and repair. From this point of view, it is clear that the mechanisms by which a histone octamer (i.e., a highly alkaline protein that packages the DNA into units called nucleosomes) binds DNA sequences, heavily impacts gene regulation at the transcriptional level at large [8], [9].

**Figure 1:**
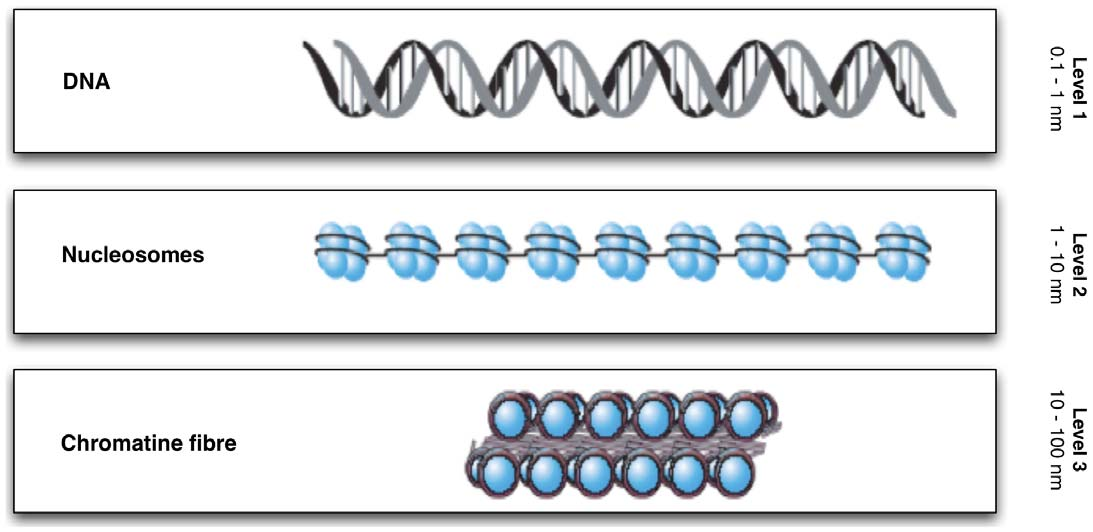
Higher order chromatin structure: level 1, the double helix DNA; level 2, DNA wrapped around histone octamers to form nucleosomes (beads on a string); level 3, nucleosomes compacted into a chromatin fiber (*i.e.*, the so-called 30 nm fiber).

The hierarchical classification for chromatin structures in interphase nuclei is divided as follows [6], [8], [10]: the linear arrangement of nucleosomes spaced by linker DNA, namely, the “nucleosomal array”, refers to the primary structure; structures formed by nucleosomal interactions, the so-called “30 nm fiber” refers to the secondary structure; structures formed by interactions between secondary structures, *i.e.*, thicker fibers seen in nuclei and postulated to be composed of 30 nm fiber refers to the tertiary structure (see Figure 1).

The chromatin structure is dubbed *euchromatin* when its wrapping is loose so that raw DNA can be accessed for transcription, otherwise it is called *heterochromatine* when the structure is a tightly packed and folded structure hence access to the polymerase is more difficult [11]. It follows that genes belonging to the same transcriptional chromatin unit can be simultaneously accessed by the polymerase. Therefore it is more likely that closely located genes on the chromosome are co-expressed, thus working in concert for implementing a certain biological function.

Starting from the well-known relationship between proximity of genes and coexpression we aim at extending the analysis to functional protein interaction. In other words, the goal of the present work is to evaluate whether proteins coded by neighboring genes are closely located in the Protein-Protein Interaction network (PPI).

We investigate this issue on the genome of the *S. cerevisiae*, a species of budding yeast that is one of the most studied organisms because of its high DNA homology with the human genome [12].

The yeast genome is composed of about 12 millions base pairs (bp) and has 6275 genes organized on 16 chromosomes (*S. cerevisiae* genome, build 2.1) although only about 5800 of them are believed to be true functional genes. A huge amount of data about *S. cerevisiae* is freely available in biological databases. In particular, the corresponding protein-protein interaction network (PPI) is an extremely well annotated network and the most used to infer topological properties [13]–[15]. In order to compare the results and to deal with the high rate of false positives and negatives we downloaded two PPI networks: MINT and BIOGRID. They are considered to be extremely valuable interactomes and largely used in network analysis.

After defining a measure of distance between genes on chromosomes we defined another measure of distance between nodes in the protein network and finally we calculated the correlation between these two measures of distance by using the information theory.

## Materials and Methods

**Figure 2:**
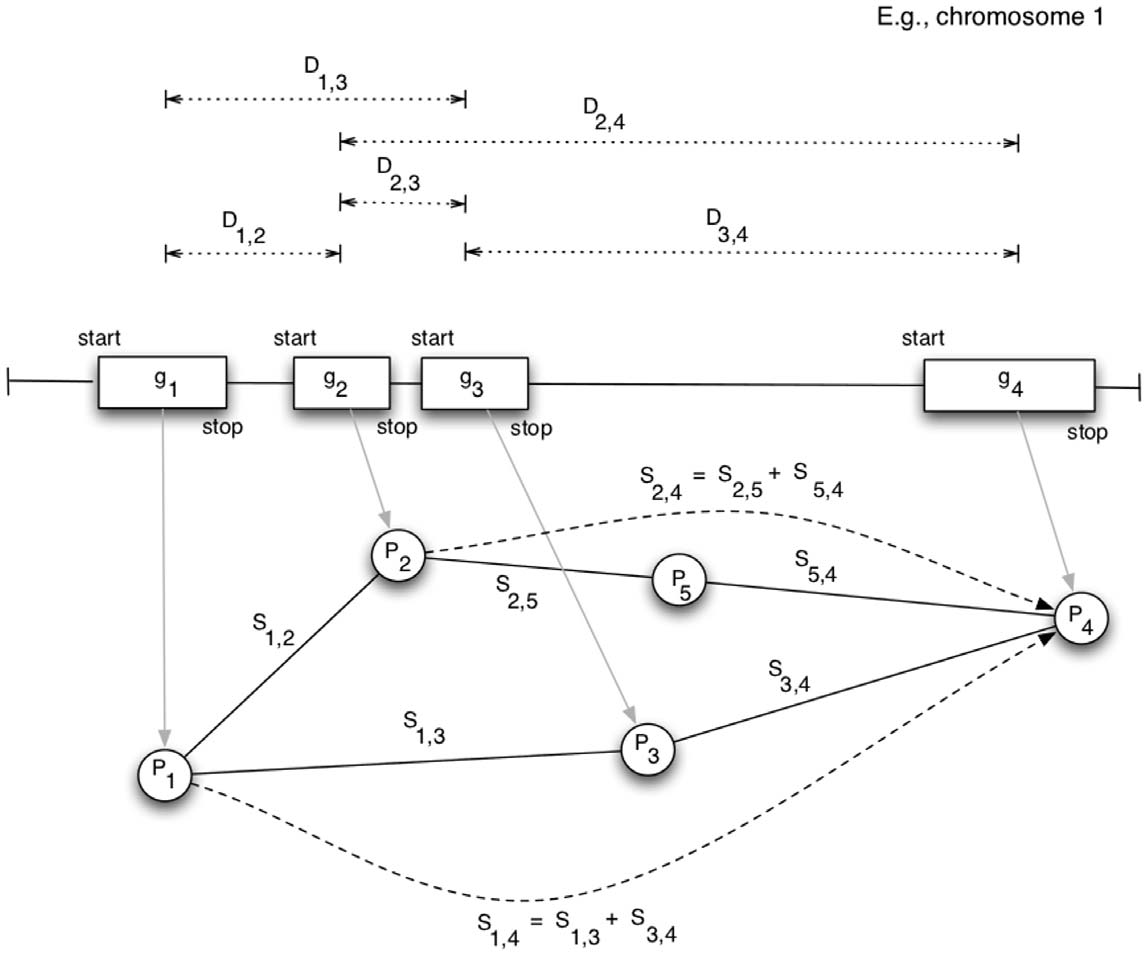
The bp-distance between genes 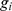 and 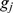 is denoted as 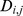. The ppi-distance is the shortest path between nodes of the corresponding PPI network indicated by 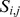. In this example the internode distance is equal to one so that 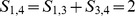 is greater than 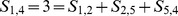.

We downloaded from NCBI (http://www.ncbi.nlm.nih.gov) the gene maps of all chromosomes of the *Saccharomyces cerevisiae* (build 2.1). Each row of these files account for information about a given gene (the start-stop position and the strand on the chromosomes, the gene name and description). For each chromosome 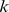, and for each couple of genes 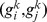, we defined the base-pair distance 
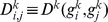
 (hereafter referred to as the *bp-distance*) as the difference of the middle position of the two genes, that is,
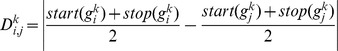
where 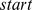 and 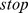 identify the start and stop base pair coordinates of genes (see Figure 2). Note that the distance 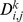 is a distance in the mathematical sense because it satisfies the usual properties of a metric: non-negativity, identity of indiscernible, symmetry and triangle inequality.

In Table 1 the length of chromosomes and the mean distance between couples of genes of the same chromosome are reported. The networks are made of 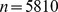 and 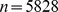 proteins for BIOGRID and MINT respectively (out of a total number of about 6300 proteins in yeast, some of the protein may have not been found involved in any interaction), with 191015 (BIOGRID) and 67228 (MINT) interactions.

**Table 1:** Size and mean gene distances on chromosomes.

	Chr 1	Chr 2	Chr 3	Chr 4	Chr 5	Chr 6	Chr 7	Chr 8
Bp size	230210	813200	316600	1531900	580000	270000	1090000	560000
Mean couple distance	66742	269069	102003	518247	182613	92628	362356	177775

	Chr9	Chr 10	Chr 11	Chr 12	Chr 13	Chr 14	Chr15	Chr 16
Bp size	440000	750000	670000	1080000	920000	780000	1090000	950000
Mean couple distance	138355	243506	207854	348330	305919	257944	352241	310755

BP size = size in base pairs = number of nucleotides.

Given a PPI we call 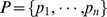 the protein set of the network and define the distance between proteins 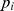 and 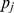 as the the shortest paths 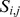 (herein referred to as the *ppi-distance*). The matrix of the shortest paths 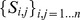 is thus composed by the shortest path of any protein couple 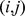 in the PPI network (see Figure 2). Note that also 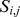 verifies the usual properties of a metric.

Now, for each chromosome 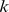, we rank all gene couples 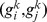 in descending order of bp-distance, and then define the following two vectors:
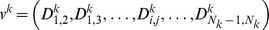


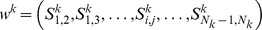
where 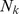 is the number of genes for each chromosome 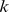 and 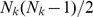 is the number of all possible couples 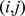. Note that since the components of 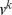 range from the smallest bp-distance on the chromosome 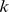 to the greatest one, then any value falls in the the interval 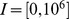 since the longest chromosome is chromosome 4 with length 1,53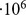. On the other hand, the shortest path distances 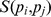 range from 1 to 5 for the BIOGRID network and from 1 to 7 for MINT. Therefore the diameter of BIOGRID is 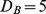 and the diameter of MINT is 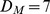.

In the following subsections we describe two different analysis performed for both networks evaluating the correlation rate between gene distances on chromosomes and PPI distances on networks. From now if not explicitly stated we mean all variables to refer to a certain chromosome 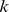, that is, we drop the superscript 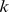.

### Analysis

Our aim is to put together two genes on the basis of the base-pair distance. In the view of the three-dimensional structure the concept of closeness may vary depending on the chromosome.

Since we could not find a linear correlation between 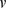 and 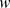 we had to resort to a finer analysis. To this purpose we resorted to the concept of mutual information which identifies a high order interdependency among variables [16]. Therefore we map the values of 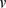 into {0,1} (0 for low distance, and 1 for high distance) by using a threshold 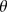, to map 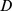 as follows
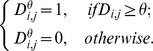
Let's call the transformed vector 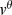 (that has components in 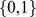),
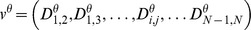
We define 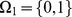 and 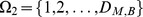 where 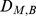 is the diameter of MINT or BIOGRID.

For a given threshold 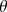, we used the mutual information (MI) defined as in [17], to measure the correlation between 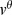 and 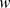


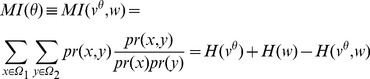
(1)where 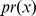 is the probability of the event 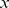 and is defined as
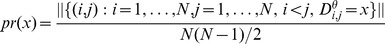


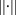
 indicates the cardinality whereas 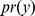 is defined as
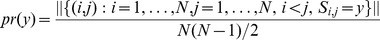
and 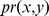 is the probability of the occurrence of couple 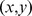 defined as
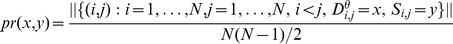
and 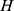 is the entropy function defined as:
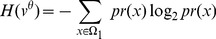
(2)

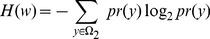
(3)and 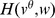 is defined as
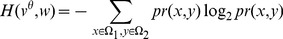
(4)In order to evaluate the deviation from randomness (*i.e.*, the statistical significance of the 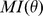) we used the Z-score function. The Z-score is a function measuringt how far a score is from the mean of the shuffle data, in unit of standard deviation. In other words the Z-score provides a p-value, identifying the probability that the observed correlation is obtained by chance [18].

To compute the Z-score we shuffle the vector 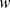, destroying any possible correlation between the two vectors 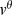 and 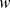 but maintaining the entropies of single vectors, respectively 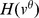 and 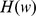. We perform 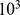 shuffles for each chromosome and each threshold and compute the related 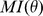 distribution. From this distribution we then compute Z-values for each 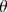 as follows:
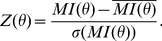
(5)


### Non-parametric statistical analysis

A further analysis was performed to highlight the correlation between bp-distance 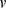 and the ppi-distance 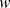 by using the Wilcoxon Test [19].

For each chromosome we defined the following vectors:
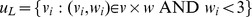


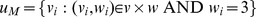
(6)

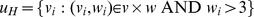
where 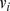 and 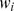 are the 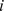-th component of the vectors 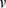 and 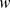. In order to evaluate whether the differences among the three sets of distances defined above are statically significant we performed a Wilcoxon test. We also computed the mean values of the three sets (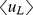, 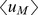 and 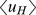).

## Results and Discussion

The major aim of this work is to provide statistical significance to the relationship between physical distance of genes on chromosomes and shortest path distance of their protein products on the PPI network. We used data from the yeast *Saccharomyces cerevisiae* interactome from two PPI networks (BIOGRID 3.1.69 and MINT) and genomic data from NCBI, build 2.1.

**Figure 3:**
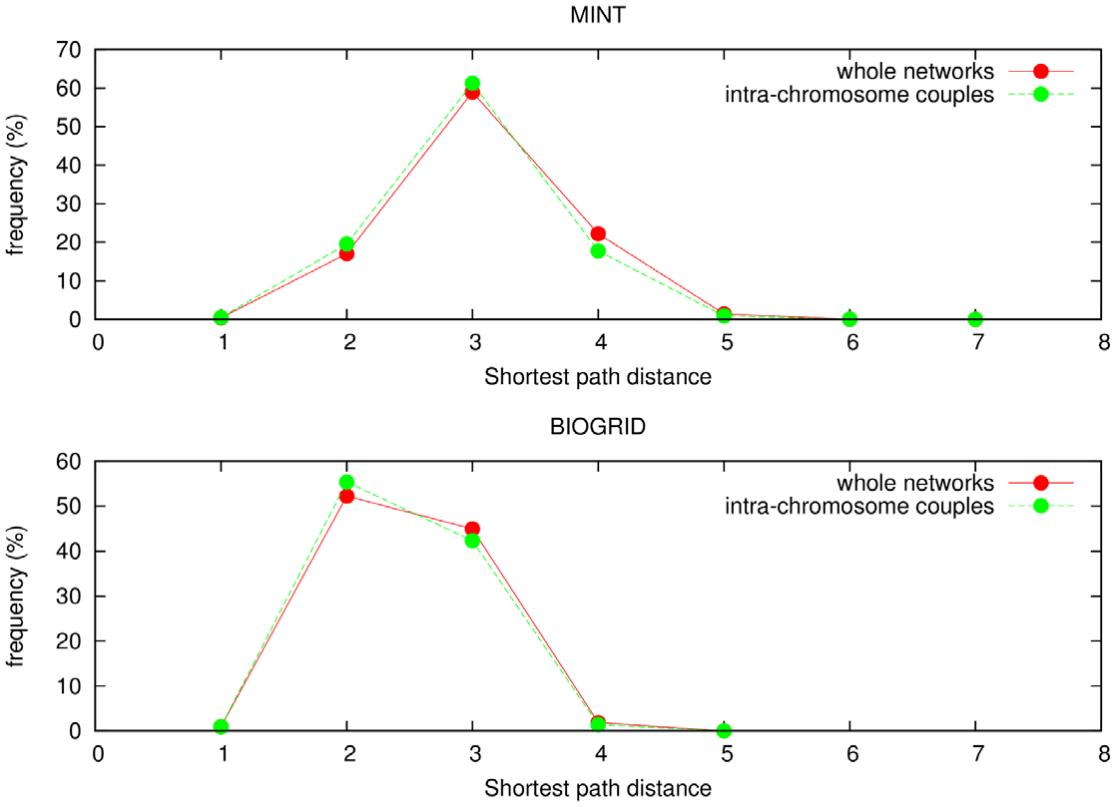
Percentage of gene couples distances for the whole network (red plot) and for the same chromosome (green plot) for both networks considered: BIOGRID (lower panel) and MINT (upper panel).

The shortest path distances range from 1 to 5 in BIOGRID and from 1 to 7 in MINT. The distribution on the whole network as well as the distribution of the shortest path distances for couple of genes belonging to the same chromosome are reported in Figure 3. Results related to BIOGRID and MINT are reported in the lower and upper panel of Figure 3 respectively.

The two distributions (whole network, red line, and intra-chromosome couples, green line), show a similar behavior in both of networks. However Figure 3 shows that the percentage of gene couples, located on the same chromosomes (green curve) with a shortest path equal to 2 is slightly greater than the one related to the whole network gene couples. On the other hand we can observe an opposite behavior for a shortest path equal to 3. Considering that the vast majority (more than 95%) of gene couples have a distance equal to 2 or 3, it suggests that it is likely that two genes, located on the same chromosomes, are closer than any other couples.

**Figure 4:**
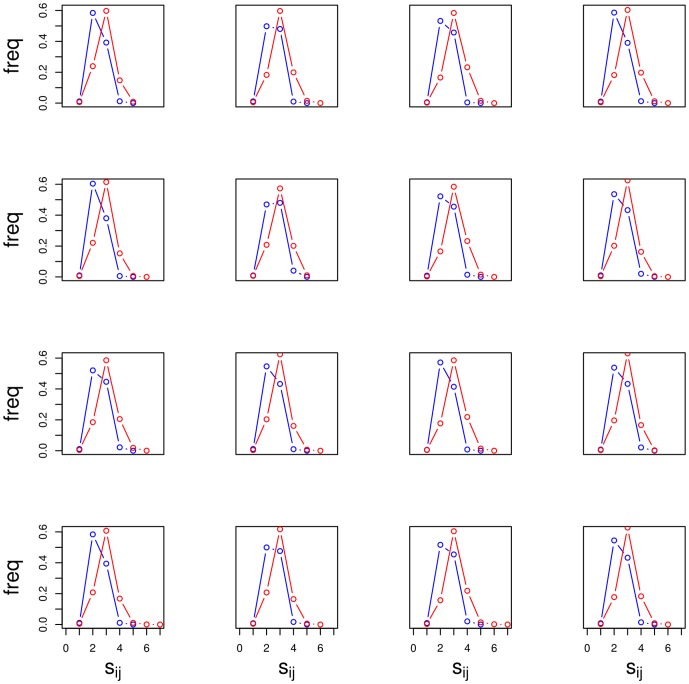
Distributions of of shortest path distances for chromosomes 1–16 (from left top to right bottom) for both networks considered: BIOGRID (blue lines) and MINT (red lines).

**Figure 5:**
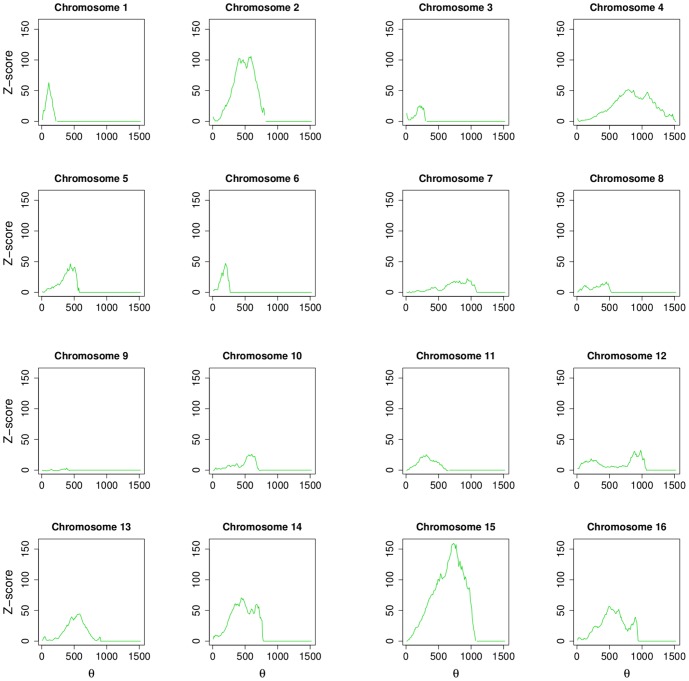
Z-score values for each chromosomes in the BIOGRID network, computed by equation 5, are reported as function of the threshold 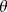
.

**Figure 6:**
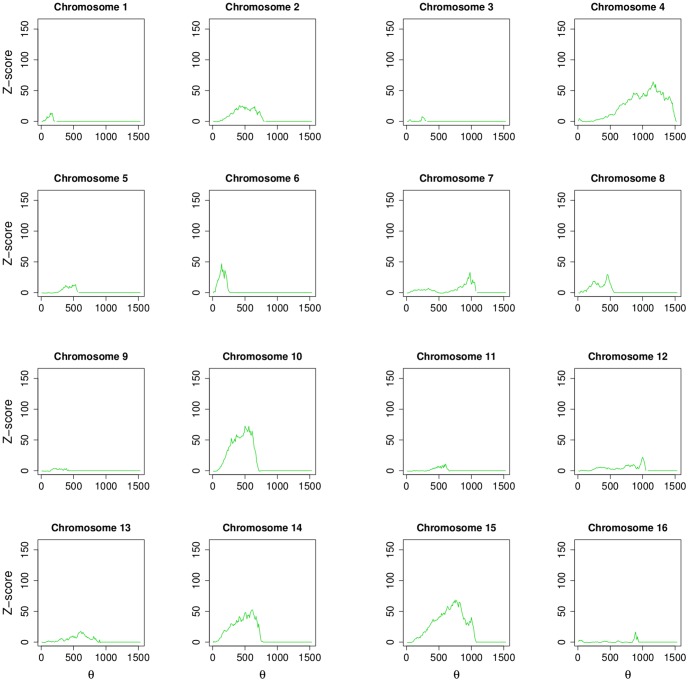
Z-score values for each chromosomes for MINT network, computed by equation 5, are reported as function of the threshold 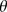
.

In Figure 4 distributions of shortest path distances for each chromosomes are shown (blue solid lines represent BIOGRID and red solid lines represent MINT). For both networks, all the profiles show a similar overall behavior. In Figure 5 (BIOGRID) and in Figure 6 (MINT) Z-score values for each chromosomes, computed by equation 5, are reported as function of the threshold 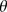. The threshold on the x-axis of Figures 5 and 6 divides couples of genes into two sets: those that are physically close and those that are far. The plots show how changes in this threshold affect the Z-score values. In other words for a given threshold value we obtain a correlation between bp-distance and sp-distance on the corresponding PPI network. A peak in the Z-score plot corresponds to the threshold value for which the correlation between bp- and sp-distance is maximal.

A read out of these plots is the following. A chromosome three-dimensional physical structure lies between the two extremes, a straight line or closely packed (*e.g.*, a ball). A single peak in the Z-score plots corresponds to a chromosome arranged in a straight line. In this case the threshold peak identifies the number of base pairs below of which two genes could interact and may be a part of the same transcriptional unit. At the other extreme (e.g., chromosome 12 in Figure 5) we find a three-dimensional structure resembling a ball. All chromosomes in both networks, with the exception of chromosome 9, show significant peaks with Z-score greater than 4 (p-value 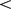
0.05), indicating that the relationship between physical distance of genes on chromosomes and shortest path distance of their protein products on the network is not driven by chance.

The large values of Z-scores obtained are due to the extremely large size of the sample. Infact, we computed the Z-score of the mutual information as the number of standard deviations from the mean of the distributions of the mutual information obtained by randomizing the sample. Since, for each chromosome, the sample is large (i.e., genes^2^ 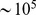), the standard deviation of MI, computed on the randomized corresponding sample, is very small. Since the Z-score is a division by this standard deviation it becomes very large. Note that this is not an artifact of the procedure: not always the relationship appears to be statistically significant for each given threshold so that the large size has the only effect to amplify the association, if any.

The most statistically significant result is obtained for chromosome 15 showing a peak around 150 standard deviation according to the definition of Z-score. Interestingly, chromosome 12 shows two clearly separated peaks around 250 kbp and 900 kbp, with a lower flat profile around the two peaks, centered in the middle of chromosome with respect to its length. This observation could be related to the strikingly different conformation of chromosome 12. In fact, in contrast to the typical pattern of intra-chromosomal interactions enveloping the lengths of entire chromosomes, chromosome 12 segregates into three distinct segments as reported by Duan et al. [20]. Regions of 430 kbp at one end and 550 kbp at the other end are engaged in extensive local interactions. These two regions do not interact with each other.

**Figure 7:**
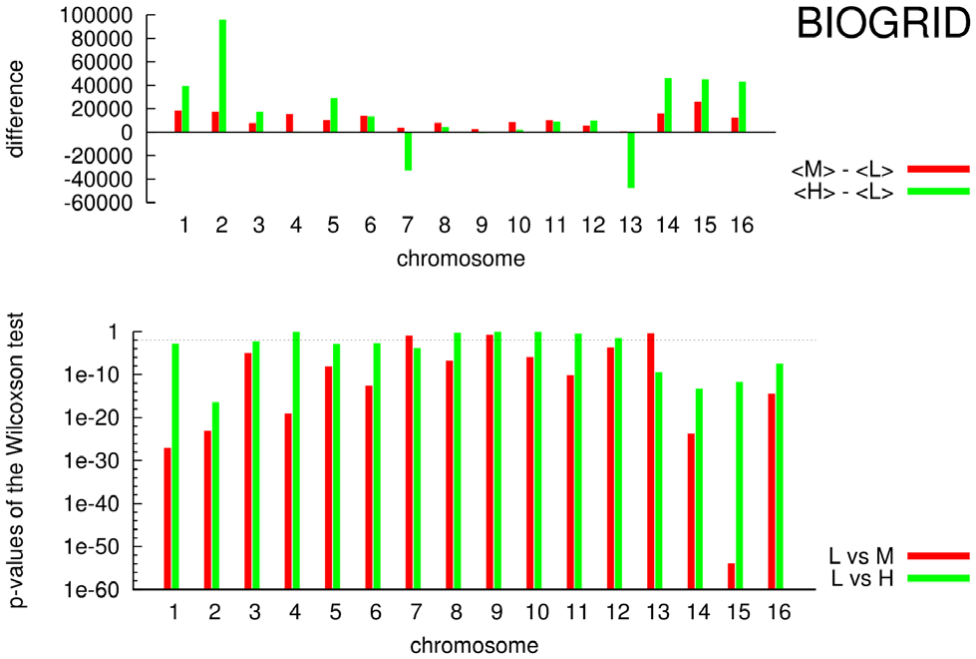
BIOGRID: for each chromosome 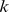, 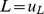, 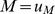 and 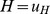 (logarithmic scale). In the lower panel horizontal dashed line represent a p-value equal to 0.05.

**Figure 8:**
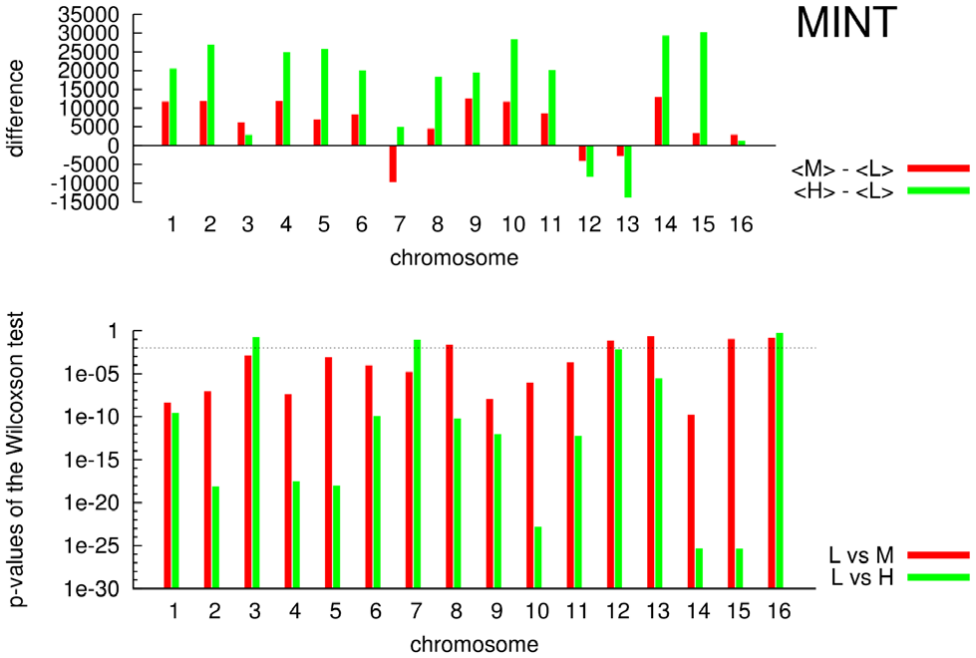
MINT: for each chromosome 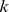, 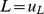, 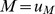 and 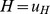 (logarithmic scale). In the lower panel horizontal dashed line represent a p-value equal to 0.05.

In the upper panels of Figures 7 (BIOGRID) and Figures 8 (MINT) are shown the differences of the mean values 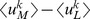 (red bars) and 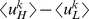 (green bars), named 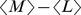 and 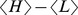 in the figures. In the lower panels of the same figures are shown the p-values obtained by Wilcoxon test for the sixteen chromosomes on the groups 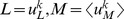 (red bars) and 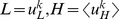 (green bars) for both of networks, BIOGRID (Figures 7) and MINT (Figures 8). In all figures (lower panels) is considered a confidence interval of 95% (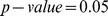) represented as dashed horizontal line. As it can be observed almost all p-values lie under the significance level revealing a statistical difference among considered groups. This finding reveals a substantial different behavior of close related genes/proteins in terms of neighborhood in the networks with respect to non related ones. In particular the mean distances of close related genes 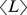 are significantly lower than the other ones 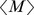 and 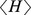. Thus close genes/proteins in the networks tend to be close as physical distance on chromosomes.

However, it is safe to stress that the positive correlation we have found does not prevent the possibility to spot counter examples. As a matter of fact, out of the set of about 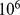 couples of genes considered (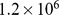 in the BIOGRID and 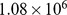 in the MINT network) we have selected few counter examples corresponding to either, i) couples of genes close on the chromosome and distant in the PPI or, ii) distant on the chromosome and close in the PPI. The selection was made by identifying two genes as close on the chromosome if within 15 k base pairs (i.e., the average gene length is around 3 k and the average inter-distance is around 10 k, so that two genes that are within 15 k bp can be, on average, considered adjacent) and distant if located at the opposite extremes of the chromosome (i.e., distance in bp greater than the 0.95 of the total length of the chromosome). On the other hand we considered two genes close in the PPI if they are directly connected (i.e., 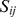 = 1) and distant those whose distance is about the network diameter). With this setting we spotted a small number of extreme-case counter examples. In particular, we identified in BIOGRID eight counter examples, four of type (i) and four of type (ii). Likewise, in MINT we identified ten counter examples, nine of type (i) and one of type (ii) (see table 2).

**Table 2:** Examples of couples of genes that do not show positive correlation between 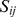 and 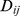 distances.

Chromosome	Gene1	Gene2	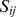	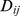	Type	Network
14	YNL270C	YNL269W	1096	5	(i)	BIOGRID
14	YNR073C	YNR075C-A	6011	5	(i)	BIOGRID
12	YLR297W	YLR301W	6953	5	(i)	BIOGRID
15	YOR337W	YOR338W	1960	5	(i)	BIOGRID
2	YBL101C	YBR296C-A	773554	1	(ii)	BIOGRID
4	YDL233W	YDR533C	1464310	1	(ii)	BIOGRID
11	YKL218C	YKR104W	639085	1	(ii)	BIOGRID
14	YNL330C	YNR069C	743206	1	(ii)	BIOGRID
3	YCR045C	YCR051W	5532	6	(i)	MINT
4	YDL109C	YDL104C	8035	6	(i)	MINT
4	YDL107W	YDL104C	4816	7	(i)	MINT
4	YDL104C	YDL102W	4254	6	(i)	MINT
4	YDL104C	YDL099W	9667	6	(i)	MINT
9	YIL166C	YIL158W	14756	6	(i)	MINT
10	YJL217W	YJL213W	9230	6	(i)	MINT
11	YKL218C	YKL217W	5310	6	(i)	MINT
12	YLR040C	YLR042C	964	6	(i)	MINT
10	YJL219W	YJR161C	723069	1	(ii)	MINT

For instance the couple YOR337W (standard name Ty Enhancer Activator 1 or TEA1) and YOR338W (which has no standard name) provides an example of genes that are close on the chromosome 15 but are distant in the BIOGRID network. Their biological processes (http://www.yeastgenome.org) associate the former gene to the cellular synthesis of RNA on a template of DNA and the latter to a positive regulation of transcription from RNA polymerase II promoter.

## Conclusions

Several works in the last decade focused on identifying and studying the conservation of proximity of certain genes in genomes as well as gene order so far. The hypothesis that chromatin structure might play a major role in the coexpression of adjacent genes is supported by diverse studies first of all Batada et al. [5] but to our knowledge no effort was made to extend the link between gene proximity and coexpression to functional interaction by Protein-Protein Interaction networks. In this article we applied concepts from information theory and non-parametric statistics in order to show that genes proximity is not driven by chance. We found that the closer the genes on chromosome, the closer their products in the PPI network, in terms of shortest paths, will be. These results support and strengthen once more the idea that chromatin structure plays a major role in the regulation process. Both mutual information and non-parametric analysis were performed separately on two different Protein-Protein Interaction networks providing in both cases the same overall behavior. This shows that the highlighted relationship between gene proximity and PPI topology is not dependent on the considered network lending robustness and generality to our results.
